# Phase Transitions Equilibria of Five Dichlorinated Substituted Benzenes

**DOI:** 10.3390/molecules28041590

**Published:** 2023-02-07

**Authors:** Ana R. R. P. Almeida, Bruno D. A. Pinheiro, Manuel J. S. Monte

**Affiliations:** Research Centre in Chemistry (CIQUP), Institute of Molecular Sciences (IMS), Department of Chemistry and Biochemistry (DQB), Faculty of Sciences, University of Porto (FCUP), Rua do Campo Alegre, 4169-007 Porto, Portugal

**Keywords:** dichlorobenzoic acids, dichlorobenzonitriles, vapor pressures, sublimation, vaporization, fusion, heat capacities

## Abstract

This work reports an experimental study aiming to determine the thermodynamic properties of five chlorinated compounds with environmental impact. The vapor pressures of the crystalline phases of three isomers of dichlorobenzoic acid (2,4-, 2,5-, and 2,6-) and 2,6-dichlorobenzonitrile were measured at several temperatures using the Knudsen effusion technique. Another technique (a static method based on capacitance diaphragm manometers) allowed the measurement of the vapor pressures of both the crystalline and liquid phases of 2,4-dichlorobenzonitrile between 303.0 and 380.0 K. This latter technique also enabled the measurement of sublimation vapor pressures of 2,6-dichlorobenzonitrile over a larger range interval of temperatures, *T* = 328.7 and 391.8 K. The standard molar enthalpy, entropy, and Gibbs energy of sublimation (for all the compounds studied) and vaporization (for 2,4-dichlorobenzonitrile) were derived, at reference temperatures, from the experimental vapor pressure results. The temperatures and enthalpies of fusion and the isobaric heat capacities of the five crystalline-substituted benzenes were determined using differential scanning calorimetry. The contributions of the three substituents (-COOH, -CN, and -Cl) to the sublimation thermodynamic properties of the compounds studied were discussed.

## 1. Introduction

Knowledge of organic compounds’ thermodynamic properties and their systematization in a convenient format is essential for several scientific, technological, and industrial applications. The measurement and prediction of these properties have received close attention since they are indispensable for optimizing processes and applications in chemistry, biotechnology, and materials science, including developing new technical equipment, sustainable energy use, and the safety assessment, behavior, and fate of pesticides and other pollutants in the environment [1,2,3,4]. Among these properties, vapor pressure occupies a prominent role due to the diversity of thermodynamic properties determined from its knowledge at different temperatures. The quantification of this fundamental physical property is often required for the industrial separation/distillation processes [5,6,7,8], in planning and monitoring activities concerning air quality in domestic environments and workplaces [9], in estimating exposure limits to volatile toxic substances [10], and in the study of precursors for thin-film deposition—a methodology used to produce high-performance hybrid organic–inorganic materials that have appeared as the next generation in photovoltaic technology [11,12].

The volatility of a material at a given temperature is adequately described by its vapor pressure (at that temperature), which is related to the Gibbs energy of vaporization or sublimation that depends both on enthalpic and entropic factors.

Information on enthalpies of sublimation or vaporization is very important for several studies, such as:

- The conversion of the gaseous phase enthalpies of formation into the condensed phases, or vice-versa;
- Estimating enthalpies of intermolecular hydrogen bonds formed in some crystalline materials, as we have investigated before (substituted benzoic acids [13], imidazoles [14], and benzamides [15]);
- Understanding the behavior of moldy/musty contaminants in cork and improving processes for their removal [4];
- The evaluation of the thermodynamic stability of compounds used in several chemical industries such as pharmaceuticals [16], fragrances and flavors [17], and agrochemicals [18].

Furthermore, the entropic contribution to Gibbs energies of sublimation or vaporization frequently helps in rationalizing the relationships between the crystalline or liquid structures predicting the behavior of these phase transitions.

Vapor pressure data are also needed to satisfy safety regulations aimed at assessing the environmental impact of chemical compounds and granting permission for their use [19]. Together with other properties such as water solubility, vapor pressure results allow the calculation of distribution coefficients, Henry’s constants, and Gibbs energies of hydration [20], which play an important role in designing new models that aim to simulate the evolution of the distribution of pollutants in the environment, particularly pesticides.

**Figure 1:**
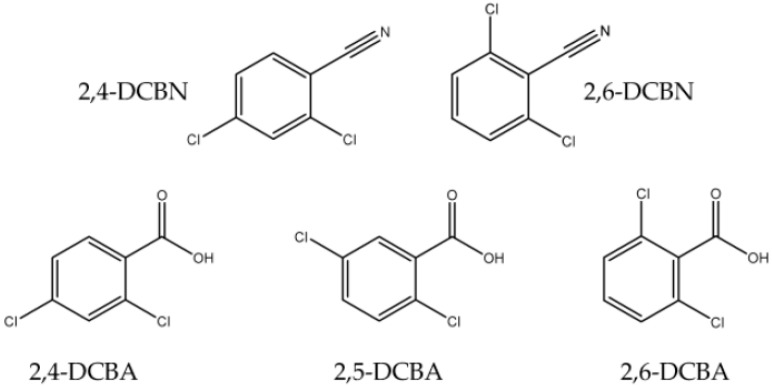
Structural formula of the compounds studied in this work.

The present work consists mainly of a volatility and phase transitions study of the herbicide dichlobenil (2,6-DCBN) and other four halogen-substituted benzenes that are used in pesticide synthesis or result in their degradation as noxious compounds: 2,4-dichlorobenzonitrile (2,4-DCBN), 2,4-dichlorobenzoic acid (2,4-DCBA), 2,5-dichlorobenzoic acid (2,5-DCBA), and 2,6-dichlorobenzoic acid (2,6-DCBA), whose structural formula is depicted in Figure 1. Dichlobenil is frequently used as a broad-spectrum contact weedkiller that has been mostly applied for controlling weeds and grasses in agricultural, industrial, and residential areas. It is also used to remove tree roots and prevent their growth in sewers [21,22,23]. Moreover, this selective and systemic herbicide is an inhibitor of the biosynthesis of cellulose and does not influence photosynthesis or cell respiration [22]. The occurrence of halogenated benzoic acids in natural environments is due predominantly to the use of herbicides and the partial degradation of spilled polychlorinated biphenyls (PCBs) [24]. 2,4-dichlorobenzoic acid is one of the degradation products of chlorfenvinphos, an organophosphorus compound, insecticide, and acaricide [25], and of the fungicide propiconazole [26]. 2,5-dichlorobenzoic acid (2,5-DCBA) is used as an intermediate in the synthesis of methyl 2,5-dichlorobenzoate [27], a plant growth regulator and antifungal agrochemical [28]. It is also involved in the production of the herbicides chloramben (3-amino-2,5-dichlorobenzoic acid) and dinoben (2,5-dichloro-3-nitrobenzoic acid) [29]. 2,6-dichlorobenzoic acid (2,6-DCBA) was reported as a plant growth regulator useful for weed control [30] and is one of the three potential metabolites produced during the degradation of the herbicide dichlobenil [31]. In addition, this compound is used as an inhibitor of an enzyme involved in breast and ovarian cancer as well as in the pathology of Alzheimer’s disease [30]. 

The thermodynamic study of these compounds is part of a project that has as its goal the experimental determination and prediction of physicochemical properties related to the environmental mobility of organic compounds with hazardous activity. Due to the absence of experimental results of thermodynamic properties for many organic compounds (due to the high cost of samples, the difficulties regarding the limitations of the experimental apparatus, or the eventual compound decomposition) and the need to screen the reliability of the data reported in the literature, several models have been proposed for predicting results, such as empirical or semi-empirical correlation models [32,33], the quantitative structure–property relationship (QSPR) [34], artificial neural networks (ANNs) [35], and also group-contribution methods [36,37,38,39]. A user-friendly group contribution model for predicting standard Gibbs energies and enthalpies of sublimation of substituted benzenes has been proposed by us in a previous study [40]. This method considers the fusion temperatures of benzene derivatives and the group contribution of their different substituents [40], as well as meaningful interactions in the ortho and para positions [40]. To enlarge the original database that supports this model, the results of more substituted benzenes have been determined or collected from the literature, allowing us to estimate the contributions of missing groups in that database, such as acetyl, acetoxy, and acetamido substituents [41,42,43]. The results of the standard Gibbs energies and enthalpies of sublimation of the five dichlorine-substituted benzenes, experimentally determined in this work, were compared with the values estimated through the predictive equations that support this model.

## 2. Results and Discussion

### 2.1. Thermodynamic Properties of Sublimation and Vaporization

Table S1 of the Supplementary Materials reports the sublimation and vaporization vapor pressures of the compounds studied in this work using the different experimental methods referred to above (Knudsen effusion and static methods). The effusion vapor pressures included in this table are the means of the results derived from the individual effusion cells at a certain temperature. The detailed experimental results obtained from each effusion cell are summarized in Table S2. The experimental (*p*,*T*) data were fit by the truncated form of the Clarke–Glew equation [44], Equation (1):

(1) $$R\ln\left(\frac{p}{p^{o}}\right)=-\frac{\Delta_{\mathrm{cd}}^{g}G_{m}^{o}\left(\theta \right)}{\theta }+\Delta_{\mathrm{cd}}^{g}H_{m}^{o}\left(\theta \right)\left(\frac{1}{\theta }-\frac{1}{T}\right)+\Delta_{\mathrm{cd}}^{g}C_{p,m}^{o}\left(\theta \right)\left[\left(\frac{\theta }{T}\right)-1+\ln\left(\frac{T}{\theta }\right)\right]$$

where *p*^o^ is a selected reference pressure (*p*^o^ = 10^5^ Pa in this work), *p* is the vapor pressure at the temperature *T*, *θ* is a reference temperature (in this work, unless stated otherwise, *θ* = 298.15 K), and *R* is the molar gas constant. The differences between gaseous and condensed phases of standard Gibbs energy, enthalpy, entropy (calculated using Equation (2)), and the isobaric heat capacity, are represented, respectively, by $\Delta_{\mathrm{cd}}^{g}G_{m}^{o}$, $\Delta_{\mathrm{cd}}^{g}H_{m}^{o}$, $\Delta_{\mathrm{cd}}^{g}S_{m}^{o}$, and $\Delta_{\mathrm{cd}}^{g}C_{p,m}^{o}$. Table 1 reports, for each compound studied, the values of these properties and their associated uncertainties for three different temperatures (*θ* = mean temperature of the experiments, *θ* = temperature of the triple point of 2,4-DCBN, and *θ* = 298.15 K). The vapor pressure results calculated from Equation (1) for the three different temperatures are also reported in this table.

(2) $$\Delta_{\mathrm{cd}}^{g}S_{m}^{o}(\theta )=\;\frac{\Delta_{\mathrm{cd}}^{g}H_{m}^{o}(\theta )-\Delta_{\mathrm{cd}}^{g}G_{m}^{o}(\theta )}{\theta }$$

#### Isobaric Heat Capacities

When accurate experimental sublimation, or vaporization, vapor pressures are measured over a wide temperature range (larger than ~50 K), the fit of Equation (1) to the experimental values frequently yields consistent values of $\Delta_{\mathrm{cd}}^{g}C_{p,m}^{o}(\theta )$. The value of $\Delta_{\mathrm{cr}}^{g}C_{p,m}^{o}(\theta )$ of the 2,6-DCBN studied in the crystalline phase, between *T* = 328.7 and 391.8 K, using the static method, and the value of $\Delta_{l}^{g}C_{p,m}^{o}(\theta )$ of the 2,4-DCBN studied in the liquid phase, between *T* = 333.3 and 380.8 K, were derived from those fittings to the (*p*,*T*) experimental results. Since the sublimation temperature ranges worked in this study using the Knudsen effusion apparatus were insufficiently large (due to the apparatus’ limitations), the values of $\Delta_{\mathrm{cr}}^{g}C_{p,m}^{o}(\theta )$ could not be calculated through the fittings referred to above. So, they were calculated as $\Delta_{\mathrm{cr}}^{g}C_{p,m}^{o}(\theta )=C_{p,m}^{o}(g)-C_{p,m}^{o}(\mathrm{cr})$, where $C_{p,m}^{o}(g)$ and $C_{p,m}^{o}(\mathrm{cr})$ are, respectively, the gas and crystalline isobaric molar heat capacities.

The values of the gas heat capacity at the temperature of 298.15 K were determined in this work for the five compounds using theoretical calculations. These results, reported in Table 2, were derived from statistical thermodynamics by applying the Gaussian 03 software package [45], using the vibrational frequencies from G3(MP2)//B3LYP calculations, scaled by a factor of (0.960 ± 0.022) [46]. For comparison reasons, the values estimated using the group contribution method proposed by Domalski and Hearing [47] are also listed in this table and are in close agreement with those calculated using our calculations.

Table 2 also includes the results of the crystal heat capacity determined in this study using differential scanning calorimetry, as well as the results estimated using the group contribution methods proposed by Domalski and Hearing [47] and by Acree Jr. and Chickos [48]. The DSC results referred to above were determined at different temperatures, enabling the fit to a third-order polynomial in temperature: $C_{p,m}^{o}(\mathrm{cr})$/J·K^−1^·mol^−1^ = a + b(*T*/K) + c(*T*^2^/K) + d(*T*^3^/K). The parameters of this equation are summarized in Table S3 of the Supplementary Materials for the five compounds studied. In all cases, the correlation coefficients were >0.999.

The value of $\Delta_{\mathrm{cr}}^{g}C_{p,m}^{o}(\theta )$/J·K^−1^·mol^−1^ = (−26.4 ± 5.5), derived from the regression of Equation (1) to the measured crystalline vapor pressure data of 2,6-DCBN, was used for the temperature adjustments of the thermodynamic properties of this compound. This result is in good agreement with the value $\Delta_{\mathrm{cr}}^{g}C_{p,m}^{o}(\theta )$/J·K^−1^·mol^−1^ = (−26.2 ± 4.8) that is derived through the difference between the results of the theoretical value of $C_{p,m}^{o}(g)$ and the result of $C_{p,m}^{o}(\mathrm{cr})$ determined in this work.

Table S4 of the Supplementary Materials includes the values of $C_{p,m}^{o}(g)$, $C_{p,m}^{o}(\mathrm{cr})$, $\Delta_{\mathrm{cr}}^{g}C_{p,m}^{o}(\theta )$, and their associated uncertainties, as well as the results of these properties estimated using various methods [49,50], namely the ones described above.

**Table 2:** Gaseous and crystalline isobaric heat capacities at constant pressure and at *T* = 298.15 K.

Method	Compound				
	2,4-DCBA	2,5-DCBA	2,6-DCBA	2,4-DCBN	2,6-DCBN
$C_{p,m}^{o}(g)$/J·K^−1^·mol^−1^					
G3(MP2)B3LYP ^a,b^	160.0 ± 4.8	160.0 ± 4.8	161.4 ± 4.8	141.6 ± 4.2	141.6 ± 4.2
Domalski and Hearing [47] ^c^	158.7 ± 4.0 ^d^	158.7 ± 4.0 ^d^	158.7 ± 4.0 ^d^	140.5 ± 4.0	140.5 ± 4.0
$C_{p,m}^{o}(cr)$/J·K^−1^·mol^−1^					
DSC (this work)	180.9 ± 2.0	179.5 ± 2.2	182.8 ± 1.8	168.9 ± 2.5	167.8 ± 2.4
Domalski and Hearing [47] ^c^	173.0 ± 4.0	173.0 ± 4.0	173.0 ± 4.0	161.1± 4.0 ^e^	161.1± 4.0 ^e^
Acree Jr. and Chickos [48] ^f^	171.5 ± 17.0	171.5 ± 17.0	171.5 ± 17.0	160.7 ± 17.0	160.7 ± 17.0

^a^ Estimated standard uncertainties: *u*($C_{p,m}^{o}(g)$/J·K^−1^·mol^−1^) = 0.03·[$C_{p,m}^{o}(g)$/J·K^−1^·mol^−1^]. ^b^ Using the scaling factor 0.96 [46]. ^c^ Group contribution method proposed by Domalski and Hearing [47]. ^d^ Calculated considering the value $C_{p,m}^{o}(g)$/J·K^−1^·mol^−1^ = 143 of 4-chlorobenzoic acid taken from the literature [51], and the group contribution values proposed by Domalski and Hearing [47]. ^e^ Calculated considering the value $C_{p,m}^{o}(\mathrm{cr})$/J·K^−1^·mol^−1^ = 165.13 for 4-nitrobenzonitrile taken from the literature [52] and the group contribution values proposed by Domalski and Hearing [47]. ^f^ Group contribution method proposed by Acree Jr. and Chickos [48].

### 2.2. Thermodynamic Properties of Fusion

The averages of the onset temperatures of fusion and the molar enthalpy and entropy of fusion of the five substituted benzenes determined using DSC (Netzsch, Selb, Germany) are reported in Table 3, together with the available literature results. Our results are in excellent agreement with the ones reported in the literature for 2,5-DCBA, 2,6-DCBA, and 2,6-DCBN. This table also describes the temperature of the triple point and the enthalpy of fusion determined indirectly using the static method for 2,4-DCBN.

### 2.3. Volatility Evaluation

**Figure 2:**
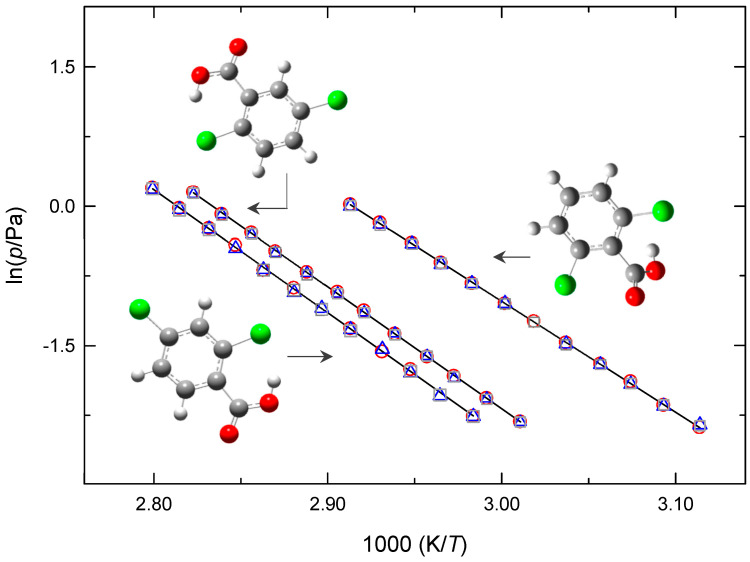
Plots of the sublimation vapor pressures (measured using the Knudsen effusion method) against reciprocal temperature for 2,4-DCBA; 2,5-DCBA, and 2,6-DCBA. ○, small effusion orifices; Δ, medium effusion orifices; □, large effusion orifices.

**Figure 3:**
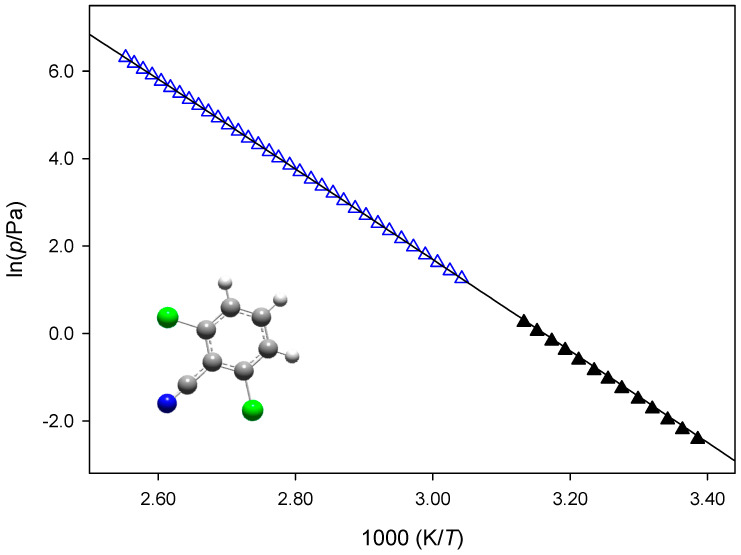
Plot of the sublimation vapor pressures against reciprocal temperature for 2,6-DCBN: Δ, MKS, static method; ▲, KEM, Knudsen effusion method.

**Figure 4:**
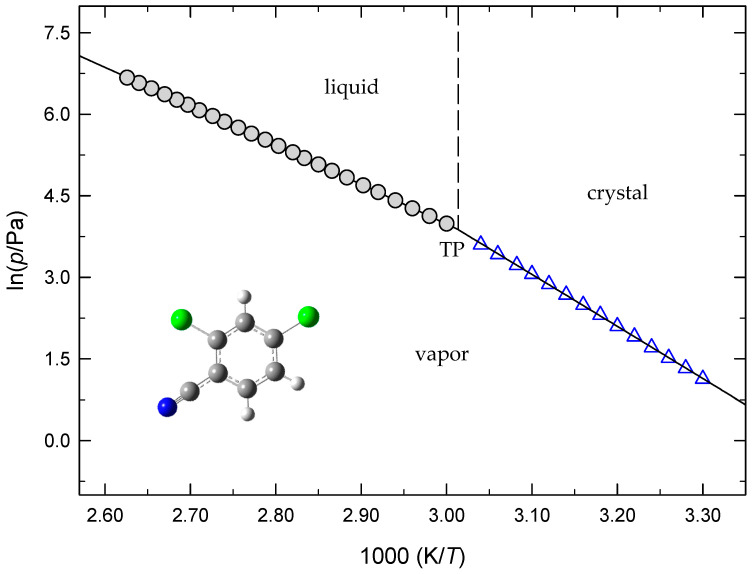
Phase diagram of 2,4-DCBN: ●, vaporization (MKS, static method); Δ, sublimation (MKS, static method); triple point (TP): *T* = 331.8 K; *p* = 48.2 Pa.

Figure 2 presents the plots of the sublimation vapor pressures against reciprocal temperatures for 2,4-DCBA, 2,5-DCBA, and 2,6-DCBA. Figure 3 shows the crystalline vapor pressures of 2,6-DCBN determined through the Knudsen effusion and static methods, and Figure 4 illustrates the phase diagram representation of the (*p*,*T*) results of 2,4-DCBN in the neighborhood of the triple point. To the best of our knowledge, no sublimation vapor pressure data have been reported before for these five compounds.

**Figure 5:**
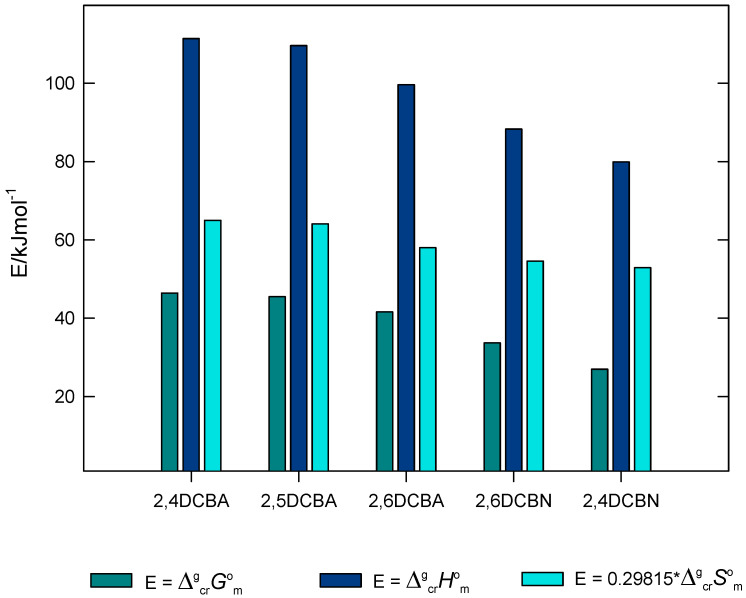
Relation between $\Delta_{\mathrm{cr}}^{g}G_{m}^{o}$, $\Delta_{\mathrm{cr}}^{g}H_{m}^{o}$, and $T\Delta_{\mathrm{cr}}^{g}S_{m}^{o}$ of the compounds studied.

The volatility of a crystalline compound at a certain temperature, *T*, decreases with increasing values in $\Delta_{\mathrm{cr}}^{g}G_{m}^{o}(T)$. Thus, considering the second member of Equation (2), the volatility decreases with increasing $\Delta_{\mathrm{cr}}^{g}H_{m}^{o}(T)$ and decreasing values of the product [*T***·**$\Delta_{\mathrm{cr}}^{g}S_{m}^{o}(Τ)$]. The contribution of these enthalpic and entropic terms for the values of $\Delta_{\mathrm{cr}}^{g}G_{m}^{o}(298.15\;K)$ of the compounds studied are easily observed in Figure 5, showing that the volatility is mostly conditioned by enthalpic factors, particularly strong for the compounds forming intermolecular hydrogen bonds. The thermodynamic properties of sublimation determined for the five compounds, reported in Table 1, and represented in Figure 5, define the following order of volatility:

2,4-DCBA < 2,5-DCBA < 2,6-DCBA < 2,6-DCBN < 2,4-DCBN

Only the crystal structures of 2,6-DCBA, 2,4-DCBA, and 2,6-DCBN were found in the literature [30,59,60]. The OH⋯O hydrogen bonds (HB) forming centrosymmetric dimers in the crystalline phases of 2,4-DCBA and 2,6-DCBA are stronger than the intermolecular interactions Cl⋯Cl or H⋯N existing in the crystals of 2,6-DCBN [60]. This explains its lower volatility compared to the studied substituted benzonitriles. Although the crystalline structures of 2,5-DCBA and 2,4-DCBN were not found in the literature, the different volatility between both compounds suggests the occurrence of OH⋯O HB dimers in the crystal pattern of the substituted benzoic acid, similarly to what happens in the crystalline packing of 2,4- and 2,6-DCBA.

**Figure 6:**
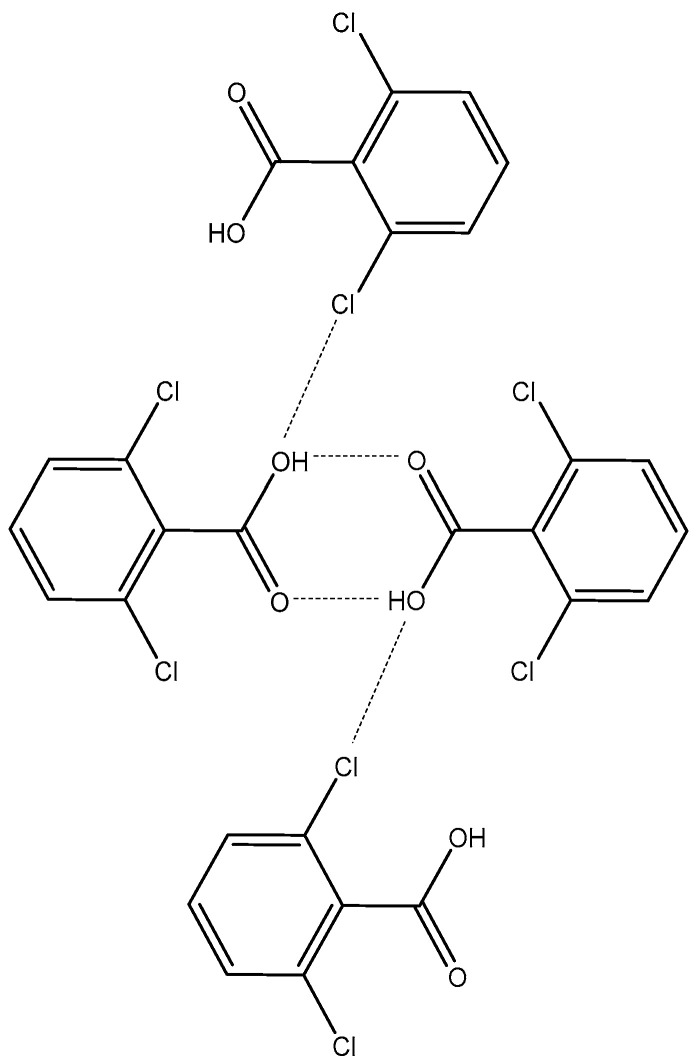
Schematic representation of the crystalline structure of 2,6-DCBA [30].

Concerning the volatility differences between the dichlorobenzoic acids investigated, it is possible to demonstrate that the presence of two chlorine atoms in ortho position in the 2,6-DCBA molecules significantly increases its volatility. The crystalline structure reported in the literature for this compound [30], represented schematically in Figure 6, suggests the establishment of intermolecular OH⋯Cl contacts (*l* = 3.233 A°) that are absent in the crystal pattern of 2,4-DCBA [59]. These interactions may be responsible for weakening the hydrogen-bonded (OH⋯O) dimers that are formed between molecules of this compound, thus boosting its volatility. The larger volatility of this isomer is due, essentially, to its smaller enthalpic contribution.

Due to the greater influence of its enthalpic factor, 2,6-DCBN is significantly less volatile than 2,4-DCBN, which means that the interactions between the 2,6 isomer molecules are stronger than those present in 2,4-DCBN. The published crystalline structure data of 2,6-DCBN indicate the formation of strong (short) intermolecular contacts between halogen atoms X⋯X and and H⋯N [60], which do not exist in other chlorinated benzonitriles such as 2-chlorobenzonitrile or 2,4,6-trichlorobenzonitrile, where the dominant intermolecular interactions are formed between the halogen atom and the nitrile group X⋯N [61,62]. However, since the 2,4-DCBN structure was not found in the literature, there is not enough information to draw a firm conclusion about the type and strength of the intermolecular interactions between its molecules.

### 2.4. Estimation of Sublimation Properties of Substituted Benzenes

A previously reported method for predicting standard Gibbs energies and enthalpies of sublimation of substituted benzenes, which considers the influence of 30 different substituent groups, is described by Equations (S2) and (S3), respectively [40]. The influence of each substituent was initially estimated, considering the sublimation results of the parameters of ca. 240 substituted benzenes. This database has been updated introducing experimental sublimation results of more benzene derivatives that confirmed those predictive equations. It already contains about 300 compounds including three new substituent groups that were absent in the original database (acetamido, acetoxy, and acetyl groups [41,42,43]). The estimation equations and the meaning of their parameters are described in the Supplementary Materials. The results of $\Delta_{\mathrm{cr}}^{g}G_{m}^{o}(298.15\;K)$ and $\Delta_{\mathrm{cr}}^{g}H_{m}^{o}(298.15\;K)$ of the five compounds studied, estimated using Equations (S2) and (S3) (described in the Supplementary Materials), respectively, are in very good agreement with those determined in this work through the vapor pressure measurements. The experimental and estimated results are reported in Table S5 of the Supplementary Materials.

## 3. Experiment

### 3.1. Materials and Purity Control

Details on the degree of purity, the purification and analysis methods, and the water content of the five compounds studied are reported in Table S6 (Supplementary Materials).

The degree of purity of the purified samples (after sublimation under reduced pressure) was checked by gas–liquid chromatography, performed using an Agilent 4890D chromatograph (Santa Clara, CA, USA), equipped with a non-polar capillary HP-5 column and a flame ionization detector (FID). Dimethylformamide was the solvent, and nitrogen was the carrier gas. The final mass fraction purities of the compounds studied were ≥0.999.

Karl Fischer’s coulometric titration was employed to determine the water content of the samples using a Methrom titration system (Herisau, Switzerland) that comprises an 831 Coulometer equipped with a generator electrode (without a diaphragm) and using HYDRANAL™ (Honeywell, Fluka) as the reagent.

### 3.2. Thermal Analysis

#### 3.2.1. Differential Scanning Calorimetry

##### Fusion Properties

To check the absence of eventual phase transitions in the compounds’ crystalline phase and determine their temperatures (onset) and enthalpies of fusion, the heat flux Netzsch calorimeter (model 204 F1 Phoenix, Selb, Germany) was used. This equipment is outfitted with a τ-sensor and an intracooler system capable of operating in the temperature range 193−873 K. Four independent runs were carried out for each compound, with samples sealed in hermetically sealed aluminum crucibles (*concavus* Al, 40 μL, Ø 5 mm, Netzsch, Selb, Germany). The samples were scanned at 2.0 K·min^−1^ from *T*/K = 298 to a temperature 15 to 20 K higher than their fusion temperature; different scans were performed through two heating–cooling cycles. Controlled nitrogen fluxes were used as a purge and as a protective gas (40 cm^3^·min^−1^ and 20 cm^3^·min^−1^), respectively, preventing the calorimeter contamination in the event of crucible hermeticity failure.

The temperature and heat flow scales of the calorimeter were calibrated using four substances from Netzsch’s kit [63]: indium (I-2803), tin (S-2776), bismuth (B-3067), and adamantane (A-2838), and using another six high-purity reference materials [64,65,66,67,68]: benzoic acid, *o*-terphenyl, naphthalene, cyclohexane, triphenylene, and biphenyl. The degree of purity of these calibrants, and the values of the enthalpy and temperature of fusion, are reported in Table S7 (Supplementary Materials). The standard uncertainties derived from the calibration results are *u*(*T*/K) = 0.46 and *u*($\Delta_{\mathrm{cr}}^{l}H_{m}^{o}(T_{\mathrm{fus}})$/kJ·mol^−1^) = 0.21.

Before melting, no crystalline phase transitions or signals of decomposition were observed under the experimental conditions used. The onset temperatures of fusion, *T*_fus_, and the molar enthalpies $\Delta_{\mathrm{cr}}^{l}H_{m}^{o}(T_{\mathrm{fus}})$ and entropies $\Delta_{\mathrm{cr}}^{l}S_{m}^{o}(T_{\mathrm{fus}})$ of fusion of the five compounds studied are reported in Table S8, together with the available literature results.

##### Crystalline Heat Capacities

Using Netzsch’s procedures and recommendations, in accordance with the ASTM E 1269, DIN 51 007, and ISO 11357-4 standards, the crystalline heat capacity measurements were performed in the present work in the temperature range of 286 to 370 K for 2,4-DCBA, 2,5-DCBA, 2,6-DCBA, and 2,6-DCBN, and between 282 and 317 K for 2,4-DCBN. Each heat capacity measurement involves three runs: blank, calibration (sapphire), and sample studied. The temperature program used contained an isothermal step at the initial temperature (25 min), a temperature ramp at 10 K⋅min^–1^, and a second isothermal step at the final temperature (25 min), using nitrogen as purge and protective gas, at flow rates of 30 cm^3^·min^−1^ and 50 cm^3^·min^−1^, respectively.

To check the reliability of the experimental technique, heat capacity experiments were performed using benzoic acid (NIST, 0.99996) and synthetic sapphire (α-Al_2_O_3_, 0.9999, disk of 0.25 mm diameter, NETZSCH) as reference material, in the temperature interval *T*/K = 281–370. The relative percentage error of our calibration measurements in comparison with those reported in the literature [69] is less than 2%. The comparison of the molar heat capacities of benzoic acid with the reference data reported in the literature [69] is shown in Figure S1 of the Supplementary Materials. All samples were placed in the aluminum crucibles (referred to above) with their lids on (unsealed) and were weighed (8–16 mg) with a precision of ±0.1 μg on a Mettler Toledo balance (model UMT2, Columbus, Ohio, United Sates).

### 3.3. Vapor Pressure Measurements

#### 3.3.1. Knudsen Mass-Loss Effusion Method

The vapor pressures of the crystalline phases of the dichlorinated benzoic acids and of 2,6-DCBN were measured at different temperatures using a Knudsen mass-loss effusion apparatus that allows the operation of nine effusion cells simultaneously. The setup and procedure of the effusion technique have been detailed, explained, and tested before [70].

During a typical experiment, the effusion cells (with different areas of the effusion orifices) are introduced in cylindrical holes inside three aluminum blocks, which are maintained at a constant temperature (that is different for the three blocks). The dimensions of the effusion orifices [71], made on platinum foil of 0.0125 ± 0.001 mm thickness, are specified in the Supplementary Materials.

For each effusion experiment, the mass loss of the samples due to the effusion process, *m*, was determined by weighing the respective effusion cells before and after the effusion period, *t*, with an estimated uncertainty of 1 × 10^−5^ g. At the temperature *T*, the vapor pressure *p* of the crystalline sample contained in each effusion cell was calculated using Equation (3):

(3) $$p=\;\frac{m}{A_{o}w_{o}t}{\left(\frac{2\pi RT}{M}\right)}^{0.5}$$

where *M* is the molar mass of the effusing vapor and *R* is the molar gas constant (*R* = 8.3144598 J·K^−1^·mol^−1^ [72]). The standard uncertainties of the temperatures and vapor pressure measurements were estimated as *u*(*T*/K) = 0.01 and *u*(*p*/Pa) = 0.02.

#### 3.3.2. Static Method Based on Capacitance Diaphragm Manometers

The vapor pressures of both the crystalline and liquid phases of 2,4-DCBN and of the crystalline phase of 2,6-DCBN, over a wider temperature interval than the one determined using the Knudsen effusion method, were measured using a static apparatus based on capacitance diaphragm manometers, that was tested and fully described before [73]. The measurements were taken using an MKS Baratron diaphragm capacitance manometer (model 631A11TBFP) operating at a self-controlled temperature (*T*_gauge_ = 473 K), which is suitable for measuring pressures in the range of 3 to 1.3 × 10^3^ Pa [74]. The expression *U*(*p*/Pa) = 0.1 + 0.0050 (*p*/Pa) describes the expanded uncertainty (0.95 confidence level, *k* = 2) of the pressure results, and the standard uncertainty of the temperature measurements is estimated to be *u*(*T*/K) = 0.01. Prior to the vapor pressure measurements, the samples were fully degassed under reduced pressure inside the apparatus, allowing for the eventual removal of any trace of volatile impurities.

## 4. Conclusions

Relevant thermodynamic properties of phase transitions of 2,4-, 2,5-, and 2,6-dichlorobenzoic acids as well as of 2,4- and 2,6-dichlorobenzonitriles were determined in this work.

- The temperatures and molar enthalpies of fusion of the five compounds studied and their crystalline isobaric molar heat capacities were determined using DSC.
- The enthalpies, entropies, and Gibbs energies of sublimation of all the compounds and of the vaporization of 2,4-DCBN were derived through vapor pressure measurements (at different temperatures) and the phase diagram representation of the (*p*,*T*) results of the latter compound, including its triple point coordinates, were reported.
- The evaluation of the enthalpic and entropic contributions to the volatility of the compounds studied was discussed.
- The contributions of -COOH, -CN, and -Cl substituents to the sublimation properties of the substituted benzenes studied were confirmed accordingly to our estimation model.

## Figures and Tables

**Table 1:** Standard (*p*^o^ = 10^5^ Pa) thermodynamic properties of sublimation of all the compounds studied and of vaporization of 2,4-DCBN.

Δ*T*	*θ*	$\Delta_{\mathrm{cd}}^{g}G_{m}^{o}\left(\theta \right)$ ^a^	*p* ^b^	$\Delta_{\mathrm{cd}}^{g}H_{m}^{o}\left(\theta \right)$ ^a^	$\Delta_{\mathrm{cd}}^{g}S_{m}^{o}\left(\theta \right)$ ^c^	*R* ^2^	$-\Delta_{\mathrm{cd}}^{g}C_{p,m}^{o}\left(\theta \right)$ ^a^	*σ*_r_^d^
K	K	kJ·mol^−1^	Pa	kJ·mol^−1^	J·K^−1.^mol^−1^		J·K^−1·^mol^−1^	
2,4-DCBA								
Crystalline phase (Knudsen effusion method)								
335.2 to 357.2	298.15	46.42 ± 0.17	7.37 × 10^−4^	111.4 ± 1.2	217.9 ± 4.1	0.9997	20.9 ± 5.2 ^e^	0.0143
	346.19 ^f^	36.03 ± 0.02	0.366	110.4 ± 1.2				
2,5-DCBA								
Crystalline phase (Knudsen effusion method)								
332.1 to 354.3	298.15	45.49 ± 0.10	1.07 × 10^−3^	109.6 ± 0.8	215.0 ± 2.7	0.9999	19.5 ± 5.3 ^e^	0.0093
	343.20 ^f^	35.86 ± 0.02	0.348	108.7 ± 0.8				
2,6-DCBA								
Crystalline phase (Knudsen effusion method)								
321.1 to 343.3	298.15	41.56 ± 0.07	5.24 × 10^−3^	99.6 ± 0.7	194.7 ± 2.4	0.9999	21.4 ± 5.1 ^e^	0.0088
	332.20 ^f^	34.97 ± 0.01	0.317	98.8 ± 0.7				
2,6-DCBN								
Crystalline phase (Knudsen effusion method)								
295.3 to 319.2	298.15	33.73 ± 0.02	1.23 × 10^−1^	88.3 ± 0.6	183.0 ± 2.0	0.9999	26.4 ± 5.5 ^g^	0.0095
	307.27 ^f^	32.06 ± 0.01	0.355	88.0 ± 0.6				
Crystalline phase (static method)								
328.7 to 391.8	298.15	33.51 ± 0.06	1.35 × 10^−1^	87.3 ± 0.7	180.4 ± 2.4	1.0000	26.4 ± 5.5 ^h^	0.0046
	360.24 ^f^	22.47 ± 0.01	55.2	85.7 ± 0.1				
2,4-DCBN								
Crystalline phase (static method)								
303.0 to 329.0	298.15	27.00 ± 0.04	1.86	79.9 ± 0.6	177.4 ± 2.0	0.9998	27.3 ± 4.9 ^e^	0.0110
	315.99 ^f^	23.85 ± 0.02	11.4	79.4 ± 0.6				
	331.83 ^i^	21.07 ± 0.04	48.2	79.0 ± 0.6	174.6 ± 0.9			
Liquid phase (static method)								
333.3 to 380.8	298.15	25.29 ± 0.04	3.71	63.7 ± 0.5	128.8 ± 1.7	1.0000	65.8 ± 8.5 ^h^	0.0036
	357.08 ^f^	18.05 ± 0.01	229	59.8 ± 0.1				
	331.83 ^i^	21.07 ± 0.03	48.2	61.5 ± 0.2	121.8 ± 0.6			

^a^ Uncertainties are expressed as the expanded uncertainty (0.95 level of confidence, *k* = 2). ^b^ Calculated from Equation (1) for three different temperatures (*θ* represents the mean temperature of the experiments, or the temperature of the triple point, or the temperature 298.15 K). ^c^ Calculated using Equation (2); uncertainties calculated through the RSS method. ^d^ *σ*_r_ is the relative standard deviation of the fit, defined as $\sigma_{r}={\left[\sum_{i=1}^{n}(\ln p\;-\;\ln p{\;}_{calc}){\;}_{i}^{2}/\;(n\;-m)\right]}^{1/2}$. ^e^ Calculated as $\Delta_{\mathrm{cr}}^{g}C_{p,m}^{o}(\theta )=C_{p,m}^{o}(g)-C_{p,m}^{o}(\mathrm{cr})$. ^f^ Mean temperature. ^g^ Value derived from the fittings of Equation (1) to the (*p*,*T*) crystalline data of 2,6-DCBN, determined through the static method. ^h^ Adjustable parameter derived from the fittings of Equation (1) to the (*p*,*T*) data. Uncertainties are standard deviations of the least squares regressions. ^i^ Temperature of the triple point.

**Table 3:** Fusion properties: temperature, molar enthalpy, and molar entropy of the compounds studied.

*T*_tp_/K	*T*_fus_/K ^a^	$\Delta_{\mathrm{cr}}^{l}H_{m}^{o}(T)$^b^/kJ·mol^−1^	$\Delta_{\mathrm{cr}}^{l}S_{m}^{o}(T)$^b,c^/J·K^−1^·mol^−1^	Method/Ref.
2,4-DCBA				
	435.20 ± 0.89	28.19 ± 0.44 ^a^	64.8 ± 1.0	DSC/this work
2,5-DCBA				
	426.87 ± 0.89	27.54 ± 0.50 ^a^	64.5 ± 1.2	DSC/this work
	426.65			[53]
	427.65			[53]
2,6-DCBA				
	414.18 ± 0.89	14.13 ± 0.26 ^a^	34.1 ± 0.6	DSC/this work
	415 ± 2			[54]
2,6-DCBN				
	416.46 ± 0.89	26.19 ± 0.34 ^a^	62.9 ± 0.8	DSC/this work
	416.7	25.94	62.3	DSC/[55]
	417.2	26.17	62.7	DSC/[56,57]
	421.2	24.56	58.3	DSC/[58]
2,4-DCBN				
	331.46 ± 0.89	17.61 ± 0.26 ^a^	53.1 ± 0.8	DSC/this work
331.8		17.5 ± 0.4	52.8 ± 1.1	static/this work

^a^ Reported experimental uncertainties were determined from the combined standard uncertainties (which include the standard deviation of the mean of the four experimental runs and the standard uncertainty of the differential scanning calorimeter calibration) and the coverage factor *k* = 2 (0.95 level of confidence). ^b^ *T* represents the temperature of fusion or the temperature of the triple point (*T*_tp_). ^c^ Uncertainties calculated through the RSS method.

## Data Availability

The data presented in this study are available in the Supplementary Materials.

## Supplementary Materials

The following supporting information can be downloaded at: https://www.mdpi.com/article/10.3390/molecules28041590/s1: Source, purity, and methods of purification and analysis of the compounds studied. Reference materials used in the calibration of the DSC calorimeter. DSC results: temperatures, molar enthalpies, and entropies of fusion of the compounds studied. Vapor pressure results. Detailed results from each effusion orifice of the sublimation of the compounds studied. Parameters of Equation (S1) of the temperature dependence of the crystalline isobaric heat capacities of 2,4-DCBA, 2,5-DCBA, 2,6-DCBA, 2,6-DCBN, and 2,4-DCBN determined using DSC. Gaseous, crystalline, and sublimation heat capacities at constant pressure and at *T* = 298.15 K. Estimation of sublimation properties of substituted benzenes. References [40,46,47,48,49,50,51,52,53,54,55,56,57,58,63,64,65,66,67,68,69] are cited in the supplementary materials.

## Sample Availability

Samples of the compounds are not available from the authors.
