# Supplementary material for: Phase Transitions Equilibria of Five Dichlorinated Substituted Benzenes
Source: Molecules. 2023 Feb 7;28(4):1590. doi: 10.3390/molecules28041590 (PMC9963677; doi:10.3390/molecules28041590)
Supplement: Supplementary file 1 [file molecules-28-01590-s001.zip › molecules-2134953-supplementary.pdf]

molecules

MDPI

SUPPLEMENTARY MATERIAL

Phase transitions equilibria of five dichlorinated substituted benzenes

Ana R. R. P. Almeida*, Bruno D. A. Pinheiro, Manuel J. S. Monte*

Research Centre in Chemistry (CIQUP), Institute of Molecular Sciences (IMS), Department of Chemistry and Biochemistry (DQB), Faculty of Sciences, University of Porto (FCUP). Rua do Campo Alegre, 4169-007 Porto, Portugal

*Authors for correspondence: ana.figueira@fc.up.pt (A.R.R.P.A.); mjmonte@fc.up.pt (M.J.S.M.)

This Supporting Material file includes the following contents:

Vapor pressure results of 2,4-DCBA, 2,5-DCBA, 2,6-DCBA, and 2,6-DCBN.

Effusion vapor pressure results of the sublimation of 2,4-DCBA, 2,5-DCBA, 2,6-DCBA, and 2,6-DCBN.

Heat capacities of the compounds studied.

Estimation of sublimation energies of substituted benzenes

Source, purity, and methods of purification and analysis of the five compounds studied.

Reference materials used in the calibration of the DSC calorimeter.

DSC results: temperatures, molar enthalpies and entropies of fusion of the compounds studied.

References

- **Vapor pressures results**

Table S1. Vapor pressure results of 2,4-, 2,5- and 2,6- DCBA, and of 2,4- and 2,6- DCBN.[a]

T/K	p/Pa	100Δp/p[b]	T/K	p/Pa	100Δp/p[b]	T/K	p/Pa	100Δp/p[b]
2,4-DCBA								
Crystalline phase (Knudsen effusion method)[c]								
335.15	0.104	0.5	343.26	0.264	-0.1	351.26	0.646	1.3
337.31	0.130	-2.6	345.24	0.332	0.7	353.14	0.781	0.2
339.24	0.169	1.2	347.14	0.405	-0.5	355.27	0.967	-1.0
341.13	0.211	1.7	349.28	0.503	-2.3	357.23	1.207	0.8
2,5-DCBA								
Crystalline phase (Knudsen effusion method)[c]								
332.14	0.098	0.2	340.28	0.252	0.3	348.43	0.609	-1.3
334.28	0.126	-0.2	342.34	0.319	0.9	350.11	0.744	0.7
336.39	0.159	-1.5	344.14	0.391	1.2	352.22	0.913	-1.1
338.15	0.198	0.6	346.25	0.486	-0.3	354.26	1.150	0.6
2,6-DCBA								
Crystalline phase (Knudsen effusion method)[c]								
321.14	0.094	1.4	329.27	0.228	-1.2	337.24	0.538	-0.6
323.29	0.117	-1.0	331.27	0.288	0.4	339.15	0.666	0.9
325.28	0.149	0.4	333.12	0.351	0.2	341.28	0.825	0.4
327.14	0.182	0.0	335.24	0.433	-1.3	343.27	1.007	0.4
2,6-DCBN								
Crystalline phase (Knudsen effusion method)[c]								
295.34	0.089	1.3	305.32	0.284	-0.1	315.13	0.842	0.6
297.31	0.111	-0.5	307.20	0.353	0.4	317.28	1.054	0.4
299.22	0.139	-0.8	309.13	0.428	-1.9	319.20	1.291	0.7
301.23	0.178	0.3	311.30	0.546	-1.4			
303.13	0.223	0.7	313.21	0.683	0.3			
Crystalline phase (static method)								
328.71	3.51	0.2	350.32	24.78	1.0	372.13	138.6	0.8
330.57	4.20	0.3	352.30	29.08	0.4	374.10	159.2	0.1

332.58	5.07	0.2	354.26	34.15	0.3	376.10	183.7	-0.2
334.55	6.05	-0.6	356.35	40.38	-0.1	378.06	211.1	-0.4
336.51	7.24	-0.7	358.21	46.99	0.1	380.04	242.7	-0.6
338.49	8.73	0.0	360.31	55.53	0.0	381.99	277.2	-1.0
340.52	10.49	0.1	362.12	64.07	0.0	383.97	319.7	-0.6
342.45	12.44	0.0	364.18	75.19	-0.1	385.92	368.7	0.2
344.51	14.86	-0.3	366.14	87.36	-0.2	387.88	419.9	-0.2
346.39	17.46	-0.5	368.08	102.0	0.5	389.85	483.0	0.5
348.42	20.90	0.1	370.10	118.0	-0.2	391.76	550.0	0.8
2,4-DCBN								
Crystalline phase (static method)								
303.03	3.10	-0.8	312.50	8.16	0.2	322.58	21.31	0.7
304.88	3.76	-0.7	314.46	10.05	1.9	324.45	25.10	0.0
306.75	4.55	-0.8	316.46	12.06	1.0	326.78	30.66	-0.9
308.64	5.53	-0.4	318.47	14.55	0.7	328.95	36.67	-2.2
310.56	6.74	0.2	320.51	17.69	1.1			
Liquid phase (static method)								
333.33	53.72	0.7	350.88	159.7	-0.2	366.80	389.6	0.1
335.57	62.02	0.3	352.91	180.0	-0.1	368.94	432.9	-0.6
337.84	71.27	-0.6	354.61	199.6	0.4	370.77	479.1	0.1
340.14	82.59	-0.5	356.74	224.9	0.2	372.55	525.4	0.2
342.47	96.07	-0.1	358.72	252.1	0.5	374.53	581.5	0.3
344.53	108.8	-0.4	360.81	281.7	0.1	376.74	648.0	0.1
346.82	125.9	0.3	362.82	314.5	0.1	378.79	715.2	-0.1
348.93	142.2	-0.2	364.96	351.1	-0.5	380.83	789.2	-0.2

[a]The standard uncertainty of the temperature is $u(T/\mathrm{K}) = 0.01$ and the expanded uncertainty (0.95 confidence level, $k = 2$) of the vapor pressures measured using the static method is $U(p/\mathrm{Pa}) = 0.1 + 0.0050\,(p/\mathrm{Pa})$. For the effusion pressures, $u(p/\mathrm{Pa}) = 0.02$. [b]$\Delta p = p - p_{calc.}$, where p_{calc} is calculated from the Clarke and Glew, Eq. (1), with parameters given in Table 3. [c]The reported effusion pressures are the means of the values obtained using the small, medium, and large effusion orifices.

Table S2. Detailed results from each effusion orifice for the sublimation of 2,4-DCBA, 2,5-DCBA, 2,6-DCBA, and 2,6-DCBN.[a]

T/K[b]	t/s	Orifices	m/mg			p/Pa[b]				
			m_S	m_M	m_L	p_S	p_M	p_L	<p>	100Δ<p>/<p>[c]
					2,4-DCBA					
335.15	25226	A_1-B_4-C_7	5.41	6.69	8.41	0.104	0.104	0.104	0.104	0.5
337.31	25226	A_2-B_5-C_8		8.39	10.59		0.130	0.131	0.130	-2.6
339.24	25226	A_3-B_6-C_9	8.95	10.67	13.66	0.172	0.166	0.169	0.169	1.2
341.13	21250	A_1-B_4-C_7	9.06	11.56		0.208	0.214		0.211	1.7
343.26	21250	A_2-B_5-C_8	11.63	14.24	17.63	0.267	0.265	0.261	0.264	-0.1
345.24	21250	A_3-B_6-C_9		17.94	22.19		0.334	0.329	0.332	0.7
347.14	18678	A_1-B_4-C_7	15.75	18.62	23.99	0.414	0.396	0.406	0.405	-0.5
349.28	18678	A_2-B_5-C_8	18.96	23.74	29.63	0.500	0.506	0.503	0.503	-2.3
351.26	18678	A_3-B_6-C_9	24.81		35.50	0.656	0.635		0.646	1.3
353.14	14442	A_1-B_4-C_7	22.90	28.16	35.05	0.786	0.782	0.775	0.781	0.2
355.27	14442	A_2-B_5-C_8	28.35	34.72	43.25	0.976	0.967	0.959	0.967	-1.0
357.23	14442	A_3-B_6-C_9	35.15	42.95	54.27	1.214	1.200	1.207	1.207	0.8
					2,5-DCBA					
332.14	23211	A_1-B_4-C_7	4.72	5.88	7.35	0.098	0.098	0.098	0.098	0.2
334.28	23211	A_2-B_5-C_8	6.06	7.53	9.33	0.126	0.126	0.125	0.126	-0.2
336.39	23211	A_3-B_6-C_9	7.70	9.29	11.88	0.160	0.157	0.159	0.159	-1.5
338.15	19541	A_1-B_4-C_7	7.96	9.93	12.47	0.197	0.199	0.199	0.198	0.6
340.28	19541	A_2-B_5-C_8	10.12	12.62	15.63	0.252	0.254	0.250	0.252	0.3
342.34	19541	A_3-B_6-C_9	12.93	15.73	19.75	0.323	0.318	0.317	0.319	0.9
344.14	14293	A_1-B_4-C_7	11.56	14.10	17.68	0.395	0.390	0.389	0.391	1.2
346.25	14293	A_2-B_5-C_8	14.31	17.54	21.69	0.491	0.487	0.479	0.486	-0.3
348.43	14293	A_3-B_6-C_9	17.85	21.77	27.43	0.614	0.606	0.608	0.609	-1.3
350.11	11407	A_1-B_4-C_7	17.34	21.07	26.74	0.750	0.737	0.744	0.744	0.7
352.22	11407	A_2-B_5-C_8	21.12	26.13	32.46	0.916	0.917	0.907	0.913	-1.1
354.26	11407	A_3-B_6-C_9	26.79	32.37	41.02	1.160	1.140	1.150	1.150	0.6
					2,6-DCBA					
321.14	21470	A_1-B_4-C_7	4.20	5.33	6.67	0.092	0.095	0.094	0.094	1.4
323.29	21470	A_2-B_5-C_8	5.31	6.52	8.33	0.117	0.116	0.118	0.117	-1.0

325.28	21470	A_3-B_6-C_9	6.82	8.23	10.39	0.151	0.147	0.148	0.149	0.4
327.14	20269	A_1-B_4-C_7	7.80	9.59	12.00	0.183	0.182	0.182	0.182	0.0
329.27	20269	A_2-B_5-C_8	9.73	11.96	14.88	0.230	0.228	0.226	0.228	-1.2
331.27	20269	A_3-B_6-C_9	12.23	13.93	18.88	0.289		0.288	0.288	0.4
333.12	15584	A_1-B_4-C_7	11.34	14.10	17.68	0.350	0.352	0.351	0.351	0.2
335.24	15584	A_2-B_5-C_8	13.99	17.34	21.73	0.433	0.434	0.433	0.433	-1.3
337.24	15584	A_3-B_6-C_9	17.42	21.23	27.06	0.541	0.533	0.541	0.538	-0.6
339.15	11210	A_1-B_4-C_7	15.47	18.99	23.77	0.670	0.665	0.663	0.666	0.9
341.28	11210	A_2-B_5-C_8	19.24	23.47	29.06	0.836	0.825	0.813	0.825	0.4
343.27	11210	A_3-B_6-C_9	23.31	28.38	35.88	1.015	1.000	1.007	1.007	0.4
					2,6-DCBN					
295.34	25536	A_1-B_4-C_7	4.87	5.98	7.26	0.091	0.090	0.087	0.089	1.3
297.31	25536	A_2-B_5-C_8	5.94	7.26	9.26	0.111	0.110	0.112	0.111	-0.5
299.22	25536	A_3-B_6-C_9	7.46	9.08	11.58	0.140	0.138	0.140	0.139	-0.8
301.23	24631	A_3-B_6-C_9	9.05	11.17	14.38	0.177	0.177	0.181	0.178	0.3
303.13	21154	A_1-B_4-C_7	9.80	12.05	15.03	0.224	0.223	0.221	0.223	0.7
305.32	21154	A_2-B_5-C_8	12.45	15.29	19.00	0.286	0.284	0.281	0.284	-0.1
307.20	21154	A_3-B_6-C_9	15.37	18.94	23.71	0.354	0.353	0.351	0.353	0.4
309.13	14659	A_1-B_4-C_7	12.66	16.12	19.96	0.422	0.434	0.428	0.428	-1.9
311.30	14659	A_2-B_5-C_8	16.18	20.47	25.18	0.541	0.554	0.542	0.546	-1.4
313.21	14659	A_3-B_6-C_9	20.54	25.24	31.21	0.689	0.685	0.674	0.683	0.3
315.13	11163	A_1-B_4-C_7	19.13	23.66	29.34	0.845	0.845	0.835	0.842	0.6
317.28	11163	A_2-B_5-C_8	23.92	29.6	36.45	1.060	1.061	1.040	1.054	0.4
319.20	11163	A_3-B_6-C_9	28.82	36.31	44.91	1.281	1.306	1.286	1.291	0.7

[a] Results related to the small (A), medium (B) and large (C) effusion orifices are denoted, respectively, by the subscripts S, M and L. [b] Estimated uncertainties: $u(T/\mathrm{K}) = 0.01$; $u(p/\mathrm{Pa}) = 0.02$. [c] $\Delta p = \langle p \rangle - \langle p \rangle_{calc.}$, where $\langle p \rangle_{calc.}$ is calculated from the Clarke and Glew equation, Eq. (1).

NOTE:

The effusion orifices of the cells used in the Knudsen effusion apparatus have the following areas: $A_o(A_1) = A_o(A_2) = A_o(A_3) = (0.636 \pm 0.004)\ \mathrm{mm}^2$, $A_o(B_1) = A_o(B_2) = A_o(B_3) = (0.785 \pm 0.004)\ \mathrm{mm}^2$ and $A_o(C_1) = A_o(C_2) =$

$A_o(C_3)$ = (0.985 ± 0.004) mm^2; their assigned uncertainties were calculated using the RSS method. The Clausing factor of the effusion orifices were calculated as $w_o = \{1 + (l/2r)\}^{-1}$, where l is the thickness of the platinum foil and r is the radius of the orifices, yielding the results 0.986, 0.988 and 0.989 for the orifices of the series A, B and C, respectively.

- **Heat Capacities**

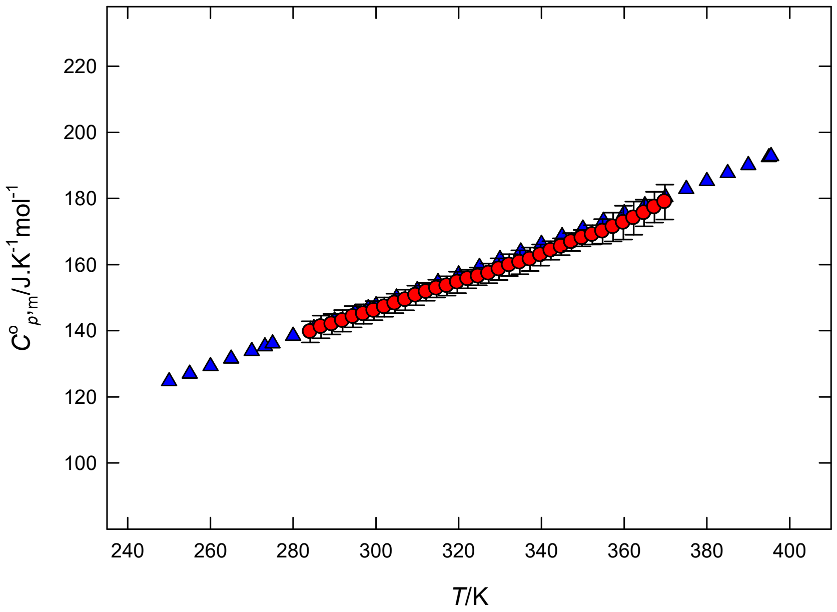

Figure S1. Comparison of the molar heat capacities, $C_{p,m}$, of benzoic acid, in the range 281 to 370 K, with the reference data reported in [1]. The error bars correspond to twice the standard deviation of six independent runs.

Table S3. Parameters of Eq. (S1) of the temperature dependence of the crystalline isobaric heat capacities of 2,4-DCBA, 2,5-DCBA, 2,6-DCBA, 2,6-DCBN and 2,4-DCBN, determined using DSC.

$$C^{o}_{p,m}(\mathrm{cr}) / \mathrm{J{\cdot}K^{-1}{\cdot}mol^{-1}} = a + b(T/\mathrm{K}) + c(T^2/\mathrm{K}) + d(T^3/\mathrm{K}) \qquad \text{(S1)}$$

Compound	a	b	c	d	$R^{2\,a}$	σ^b	Temperature range / K
2,4-DCBA	–209.33	3.0092	–8.3938×10^{-3}	9.0261×10^{-6}	0.9997	0.21	286.4 – 369.8
2,5-DCBA	–600.80	6.5064	–1.8719×10^{-2}	1.9032×10^{-5}	0.9998	0.17	286.4 – 369.8
2,6-DCBA	–403.07	4.7832	–1.3820×10^{-2}	1.4648×10^{-5}	0.9998	0.16	286.4 – 369.8

2,6-DCBN	–642.84	7.2052	–2.2149×10^{-2}	2.3821×10^{-5}	0.9998	0.13	286.4 – 369.8
2,4-DCBN	66.756	0.30356	5.3408×10^{-5}	2.5968×10^{-7}	0.9999	0.05	271.4 – 319.7

[a] Coefficient of determination; [b] Standard error of estimate.

Table S4. Gaseous, crystalline and sublimation isobaric heat capacities at constant pressure, at T = 298.15 K. All values in J·K^{-1}·mol^{-1}. (Selected values in bold)

Method	Compound				
	2,4-DCBA	2,5-DCBA	2,6-DCBA	2,4-DCBN	2,6-DCBN
			$C_{p,m}^{o}$(g)		
A. G3(MP2)B3LYP[a,b]	**160.0 ± 4.8**	**160.0 ± 4.8**	**161.4 ± 4.8**	**141.6 ± 4.2**	**141.6 ± 4.2**
B. Domalski and Hearing[c]	158.7 ± 4.0[d]	158.7 ± 4.0[d]	158.7 ± 4.0[d]	140.5 ± 4.0	140.5 ± 4.0
			$C_{p,m}^{o}$(cr)		
C. Experimental	**180.9 ± 2.0**	**179.5 ± 2.2**	**182.8 ± 1.8**	**168.9 ± 2.5**	**167.8 ± 2.4**
D. Domalski and	173.0 ± 4.0	173.0 ± 4.0	173.0 ± 4.0	[illegible]	[illegible]
E. Acree Jr. and Chickos[f]	171.5 ± 17.0	171.5 ± 17.0	171.5 ± 17.0	160.7 ± 17.0	160.7 ± 17.0
			$-\Delta_{cr}^{g}C_{p,m}^{o}$ g		
$\Delta_{cr}^{g}C_{p,m}^{o} = C_{p,m}^{o}(g) - C_{p,m}^{o}(cr)$					
A – C	**20.9 ± 5.2**	**19.5 ± 5.3**	**21.4 ± 5.1**	**27.3 ± 4.9**	**26.2 ± 4.8**
A – D	13.0 ± 6.2	13.0 ± 6.2	11.6 ± 6.2	19.5 ± 5.8	19.5 ± 5.8
A – E	11.5 ± 17.7	11.5 ± 17.7	10.1 ± 17.7	19.1 ± 17.5	19.1 ± 17.5
B – C	22.2 ± 4.5	20.8 ± 4.6	24.1 ± 4.4	28.4 ± 4.7	27.3 ± 4.7
B – D	14.3 ± 5.7	14.3 ± 5.7	14.3 ± 5.7	20.6 ± 5.7	20.6 ± 5.7
B – E	12.8 ± 17.5	12.8 ± 17.5	12.8 ± 17.5	20.2 ± 17.5	20.2 ± 17.5

$\Delta_{cr}^{g}C_{p,m}^{o} = -\{0.9 + 0.176 \cdot C_{p,m}^{o}(g)\}$ [h]					
A	29.1 ± 33.3	29.1 ± 33.3	29.3 ± 33.3	25.8 ± 33.3	25.8 ± 33.3
B	28.8 ± 33.2	28.8 ± 33.2	28.8 ± 33.2	25.6 ± 33.2	25.6 ± 33.2
$\Delta_{cr}^{g}C_{p,m}^{o} = -\{0.75 + 0.15 \cdot C_{p,m}^{o}(cr)\}$ [i]					
C	27.9 ± 33.1	27.7 ± 33.1	28.2 ± 33.1	26.1 ± 33.1	25.9 ± 33.1
D	26.7 ± 33.2	26.7 ± 33.2	26.7 ± 33.2	24.9 ± 33.2	24.9 ± 33.2
E	26.5 ± 37.1	26.5 ± 37.1	26.5 ± 37.1	24.9 ± 37.1	24.9 ± 37.1
Eq. (1) [j]					26.4 ± 5.5

[a] Estimated standard uncertainties: $u(C_{p,m}^{o}(g)/J\cdot K^{-1}\cdot mol^{-1}) = 0.03\cdot[C_{p,m}^{o}(g)/J\cdot K^{-1}\cdot mol^{-1}]$, [b] Using the scaling factor 0.96 [2]; [c] Group contribution method proposed Domalski and Hearing [3]; [d] Calculated considering the value $C_{p,m}^{o}(g)/J\cdot K^{-1}\cdot mol^{-1} = 143$ of 4-chlorobenzoic acid taken from literature [4], and the group contribution values proposed by Domalski and Hearing [3]; [e] Calculated considering the value $C_{p,m}^{o}(cr)/J\cdot K^{-1}\cdot mol^{-1} = 165.13$ for 4-nitrobenzonitrile taken from literature [5] and the group contribution values proposed by Domalski and Hearing [3]. [f] Group contribution method proposed by Acree Jr. and Chickos [6]; [g] Standard uncertainties calculated through the RSS method; [h] Proposed by Monte *et al.* [7] and by Acree Jr. and Chickos [6]; [i] Proposed by Chickos *et al.* [8]; [j] Clarke and Glew equation, Eq 1.

- **Estimation of sublimation properties of substituted benzenes**

The following equations, fully described and explained before [9], were used to estimate the energies of sublimation of the compounds studied. A brief explanation is given below:

$$\Delta_{\rm cr}^{\rm g}G_{\rm m}^{\rm o}\ (298.15\ {\rm K})/{\rm kJ\cdot mol^{-1}} = -(11.5 \pm 0.7) + (0.056 \pm 0.003)\ (T_{\rm fus}/{\rm K}) + \sum n_{\rm i}.g_{\rm i} + \sum n_{\rm x}.x^{G}{}_{({\rm R_j/R_k})} + \sum n_{y}.y^{G}{}_{({\rm R_j/R_k})}$$

(**S2**)

$$\Delta_{\rm cr}^{\rm g}H_{\rm m}^{\rm o}\ (298.15\ {\rm K})/{\rm kJ\cdot mol^{-1}} = (40.0 \pm 1.5) + (0.031 \pm 0.005)(T_{\rm fus}/{\rm K}) + \sum n_{\rm i}.h_{\rm i} + \sum n_{x}.x^{H}{}_{({\rm R_j/R_k})} + \sum n_{y}.y^{H}{}_{({\rm R_j/R_k})}$$

(**S3**)

These equations consider the contribution of the substituent groups in the benzene core of the molecules for intermolecular interactions in the crystalline phase. Eventual interactions between some of these groups in *ortho* and *para* positions were found to be significant and were considered in the above equations [9]. The contribution of each substituent *i* to [$\Delta_{\rm cr}^{\rm g}G_{\rm m}^{\rm o}$ (298.15K) – 0.056($T_{\rm fus}$/K)], and to [$\Delta_{\rm cr}^{\rm g}H_{\rm m}^{\rm o}$ (298.15 K) – 0.031($T_{\rm fus}$/K)], are designated by g_i and h_i, respectively, and n_i symbolizes its number. $x^{\rm G}{}_{\rm (Rj/Rk)}$ and $x^{\rm H}{}_{\rm (Rj/Rk)}$ refer to the contributions of the eventual interactions between the substituents *R*j and *R*k in *ortho* positions, and $n_{\rm x}$ stands for the number of these interactions; $y^{\rm G}{}_{\rm (Rj/Rk)}$ and $y^{\rm H}{}_{\rm (Rj/Rk)}$ and $n_{\rm y}$ have analogous meanings as above for substituents in *para* positions to each other. Significant interactions in *meta* position were not detected.

The original database that supports this estimation method contains the groups of the substituted benzenes studied in this work: 82 compounds substituted with -COOH, 7 compounds with -CN, and 35 with -Cl atom. The contributions assigned before for these substituent groups considered were $g_{\rm i}$ (-COOH) = (24.1 ± 0.5) kJ.mol^{-1} and $h_{\rm i}$ (-COOH) = (40.4 ± 1.0) kJ.mol^{-1}; $g_{\rm i}$ (-CN) = (11.6 ± 0.5) kJ.mol^{-1} and $h_{\rm i}$ (-CN) = (16.3 ± 1.1) kJ.mol^{-1} and $g_{\rm i}$ (-Cl) = (4.6 ± 0.2) kJ.mol^{-1} and $h_{\rm i}$ (-Cl) = (7.2 ± 0.3) kJ.mol^{-1} [9].

The comparison between the experimental results of the compounds studied and the estimated values using equations S2 e S3, are reported in Table S5.

Table S5. Experimental and estimated results of $\Delta_{cr}^{g}G_{m}^{o}$ and of $\Delta_{cr}^{g}H_{m}^{o}$, at *T* = 298.15 K, of the five dichlorinated substituted benzenes studied in this work.

Compound	$\Delta_{cr}^{g}G_{m}^{o}$		$\Delta_{cr}^{g}H_{m}^{o}$	
	Exp.	Est. (eq, S2)[a]	Exp.	Est. (eq, S3)[b]
2,4-DCBN	27.0 ± 0.1	27.9 ± 1.3	79.9 ± 0.6	81.0 ± 2.1
2,6-DCBN	33.6 ± 0.4[b]	32.6 ± 1.3	87.8 ± 0.7[b]	83.6 ± 2.1
2,4-DCBA	46.4 ± 0.2	46.2 ± 1.3	111.4 ± 1.2	108.3 ± 2.1
2,5-DCBA	45.5 ± 0.1	45.7 ± 1.3	109.6 ± 0.8	108.0 ± 2.1
2,6-DCBA	41.6 ± 0.1	41.7 ± 1.3	99.6 ± 0.7	102.0 ± 2.1

[a]Uncertainties calculated from the uncertainties described in Eqs. S2 or S3 using the root-sum-square method. [b]Mean value of the results derived through vapor pressure measurements, determined using Knudsen effusion and static methods.

- **Materials and purity control**

Table S6. Source, purity, and methods of purification and analysis of the five compounds studied.

Compound	CASNR	Source	Minimum initial purity[a]	Purification Method	Final mass fraction purity	Analysis method[b]	% Water content[c]
2,4-DCBA	50-84-0	Sigma-Aldrich	0.996	Sublimation under reduced pressure	0.9990	GC (FID)	0.02 ± 0.01
2,5-DCBA	50-79-3	Alfa Aesar	0.983		0.9993		0.03 ± 0.01
2,6-DCBA	50-30-6	Alfa Aesar	0.988		0.9982		0.03 ± 0.01
2,6-DCBN	1194-65-6	Alfa Aesar	0.990		0.9986		0.04 ± 0.01
2,4-DCBN	6574-98-7	Alfa Aesar	0.999	--	0.9988		0.03 ± 0.01

[a]As stated in the certificate of analysis from the manufacturer. [b]Gas-liquid chromatography with flame ionization detector; the results are related to a dry basis. [c]Determined using Karl Fisher coulometric titration (mass percentage). The error was assigned as the standard deviation of the mean of four independent measurements.

- **Differential scanning calorimetry**

Table S7. Reference materials used in the calibration of the DSC calorimeter.

Calibrant	Source	Initial purity[a]	Final mass fraction	T_{fus}/K	$\Delta_{cr}^{l}H_{m}^{o}(T_{fus})/$	Ref.
Benzoic acid[c]	NIST	0.99996	--	395.50 ± 0.02	17.98 ± 0.04	[10-12]
Biphenyl	Sigma	0.995	0.9988	342.08	18.57 ± 0.01	[12]

Naphthalene	Aldrich	0.99	0.9999	353.38 ± 0.02	18.92 ± 0.08	[10-12]
o-Terphenyl		0.999	0.9991	329.40	17.19 ± 0.01	[11,13,14]
Triphenylene		0.999	0.9983	471.0 ± 0.06	24.74 ± 0.01	[10,11,13]
Cyclohexane Uvasol	Merck	0.999	0.9992	279.81 ± 0.04	2.65 ± 0.07	[11]
Adamantane (A-2838)	NETZSCH	>0.99	--	208.7	3.00	[15]
Bismuth (B-3067)		0.99999	--	544.6	11.10	[15]
Indium (I-2803)		0.99999	--	429.8	3.28	[15]
Tin (S-2776)		0.99999	--	505.1	7.18	[15]

[a]Purity degree stated by the supplier. [b]Gas-liquid chromatography (flame ionization detector). [c]Standard reference material (SRM) 39j.

Table S8. DSC results: temperatures, molar enthalpies and entropies of fusion of the compounds studied.

Exp.	T_{fus}(onset)/K	$\Delta_{cr}^{l}H_{m}^{o}(T_{fus})$ /kJ·mol^{-1}	$\Delta_{cr}^{l}S_{m}^{o}(T_{fus})$ /J·K^{-1}·mol^{-1}
		2,4-DCBA	
1	435.20	27.71	
2	435.30	28.23	
3	435.27	28.61	
4	435.02	28.22	
Mean	435.20 ± 0.89[a]	28.19 ± 0.44[a]	64.8 ± 1.0[b]
		2,5-DCBA	
1	426.79	27.56	
2	426.77	27.66	
3	426.91	28.00	
4	427.02	26.95	
Mean	426.87 ± 0.89[a]	27.54 ± 0.50[a]	64.5 ± 1.2[b]
Lit. [16]	426.65		
Lit. [16]	427.65		
		2,6-DCBA	
1	414.30	14.29	
2	414.05	14.12	
3	414.17	14.09	
4	414.21	14.03	
Mean	414.18 ± 0.89[a]	14.13 ± 0.26[a]	34.1 ± 0.6[b]
Lit. [17]	415 ± 2		
		2,4-DCBN	
1	331.60	17.49	
2	331.45	17.75	
3	331.33	17.58	
4	331.44	17.65	
Mean	331.46 ± 0.89[a]	17.61 ± 0.26[a]	53.1 ± 0.8[b]
		2,6-DCBN	
1	416.29	26.20	
2	416.53	25.85	
3	416.57	26.28	
4	416.45	26.44	
Mean	416.46 ± 0.89[a]	26.19 ± 0.34[a]	62.9 ± 0.8[b]
Lit. [18]	416.7	25.94	
Lit. [19,20]	417.2	26.17	
Lit. [21]	421.2	24.56	

[a]The reported experimental uncertainties were determined from the combined standard uncertainties (which include the standard deviation of the mean of the four experimental runs and the standard uncertainty of the differential scanning calorimeter calibration) and the coverage factor k = 2 (0.95 level of confidence). [b]Uncertainties calculated through the RSS method.

References

[1] Furukawa, G.T.; McCoskey, R.E.; King. G.J. Calorimetric properties of benzoic acid from 0° to 410° K. *J. Res. Natl. Inst.* **1951**, *47*, 256–261.

[2] NIST Computational Chemistry Comparison and Benchmark Database, NIST Standard Reference Database Number 101, Release 16a; Johnson, R.D., III, Ed.; NIST Chemistry Web Book, 2013. SRD 69. Available online: http://cccbdb.nist.gov/vibscalejust.asp (accessed on 2 December 2022).

[3] Domalski, E.S.; Hearing, E.D. Estimation of the thermodynamic properties of C-H-N-O-S-halogen compounds at 298.15 K. *J. Phys. Chem. Ref. Data* **1993**, *22*, 805–1159.

[4] Monte, M.J.S.; Santos, L.M.N.B.F.; Fonseca, J.M.S.; Sousa, C.A.D. Vapour pressures, enthalpies and entropies of sublimation of para substituted benzoic acids. *J. Therm. Anal. Calorim.* **2010**, *100*, 465–474.

[5] Ribeiro da Silva, M.A.V.; Monte, M.J.S.; Rocha, I.M.; Cimas Á. Energetic study applied to the knowledge of the structural and electronic properties of monofluorobenzonitriles. *J. Org. Chem.* **2012**, *77*, 4312–4322.

[6] Acree, W., Jr.; Chickos, J.S. Phase transition enthalpy measurements of organic and organometallic compounds. Sublimation, vaporization and fusion enthalpies from 1880 to 2015. Part 1. C1-C10. *J. Phys. Chem. Ref. Data* **2016**, *45*, 033101.

[7] Monte, M.J.S.; Almeida, A.R.R.P.; Matos; M.A.R. Thermodynamic study on the sublimation of five aminomethoxybenzoic acids *J. Chem. Eng. Data* **2010**, *55*, 419−423.

[8] Chickos, J.S.; Hosseini, S.; Hesse, D.G.; Liebman, J.F. Heat capacity corrections to a standard state: a comparison of new and some literature methods for organic liquids and solids. *Struct. Chem.* **1993**, *4*, 271–278.

[9] Monte, M.J.S.; Almeida, A.R.R.P. A new approach for the estimation of sublimation enthalpies and vapor pressures of crystalline benzene derivatives. *Struct. Chem*. **2013**, *24*, 2001−2016.

[10] Sabbah, R.; El. Watik, L. New reference materials for the calibration (temperature and energy) of differential thermal analysers and scanning calorimeters. *J. Therm. Anal.* **1992**, *38*, 855−863.

[11] Sabbah, R.; Xu-Wu, A.; Chickos, J.S.; Planas Leitão, M.L.; Roux, M.V.; Torres, L.A. Reference materials for calorimetry and differential thermal analysis. *Thermochim. Acta* **1999**, *331*, 93−204.

[12] Della Gatta, G.; Richarson, M.J.; Sarge, S.M.; Stølen, S. Standards, calibration, and guidelines in microcalorimetry part 2. Calibration standards for differential scanning calorimetry. *Pure Appl. Chem*. **2006**, *78*, 1455−1476.

[13] Roux, M.V.; Temprado, M.; Chickos, J.S.; Nagano, Y.J. Critically evaluated thermos chemical properties of polycyclic aromatic hydrocarbons. *Phys. Chem. Ref. Data* **2008**, *37*, 1855−1996.

[14] Chang, S.S.; Bestul, A.B. Heat capacity and thermodynamic properties of o-terphenyl crystal, glass, and liquid *J. Chem. Phys*. **1972**, *56*, 503−516.

[15] NETZSCH Analyzing & Testing DSC 204 F1 Phoenix. Calibration set. **2022**, Selb, Germany. Available online: https://analyzing-testing.netzsch.com/_Resources/Persistent/3/f/6/2/3f62fce9e4fd19c3cb5d75f1514a90ef444f9c23/DSC_204_F1_Phoenix_en_web.pdf (accessed on 14 December 2022)

[16] Altau, K.; Beasley, J.G.; Pine, H.J.; Crawford, R.H.; Tucker, W.T.; Brown, A.D.; Capps, J.D. *J. Chem. Eng. Data* **1963**, *8*, 122 Available online: https://webbook.nist.gov/cgi/cbook.cgi?ID=C50793&Units=SI&Mask=4#Thermo-Phase (accessed on 21 November 2022)

[17] Stork, G.; White, W.N. *J. Am. Chem. Soc*. **1956**, *78*, 4609. Available online: https://webbook.nist.gov/cgi/cbook.cgi?ID=C50306&Units=SI&Mask=4#ref-1 (accessed on 21 November 2022)

[18] Plato, C. Differential scanning calorimetry as a general method for determining purity and heat of fusion of high-purity organic chemicals. Application to 64 compounds. Anal. Chem. 1972, 44, 1531–1534.

[19] Donnelly, J.R.; Drewes, L.A.; Johnson, R.L.; Munslow, W.D.; Knapp, K.K.; Sovocool, G.W. Purity and heat of fusion data for environmental standards as determined by differential scanning calorimetry. Thermochim. Acta 1990, 167, 155–187.

[20] Acree, W.E., Jr. Thermodynamic properties of organic compounds: enthalpy of fusion and melting point temperature compilation. *Thermochim. Acta* **1991**, *189*, 37–56, references therein.

[21] Rodante, F.; Vecchio, S.; Catalani, G.; Guidotti, M. Thermal analysis and non-isothermal kinetic study of some pesticides. Part II. Chlorinate derivatives. *J. Therm. Anal. Calorim*. **2000**, *60*, 605–622.
